# New CXCR4 Antagonist Peptide R (Pep R) Improves Standard Therapy in Colorectal Cancer

**DOI:** 10.3390/cancers12071952

**Published:** 2020-07-18

**Authors:** Crescenzo D’Alterio, Antonella Zannetti, Anna Maria Trotta, Caterina Ieranò, Maria Napolitano, Giuseppina Rea, Adelaide Greco, Piera Maiolino, Sandra Albanese, Giosuè Scognamiglio, Fabiana Tatangelo, Salvatore Tafuto, Luigi Portella, Sara Santagata, Guglielmo Nasti, Alessandro Ottaiano, Roberto Pacelli, Paolo Delrio, Gerardo Botti, Stefania Scala

**Affiliations:** 1 Research Department, Microenvironment Molecular Targets, Istituto Nazionale Tumori IRCCS “Fondazione G. Pascale”, 80131 Napoli, Italy; c.dalterio@istitutotumori.na.it (C.D.); am.trotta@istitutotumori.na.it (A.M.T.); c.ierano@istitutotumori.na.it (C.I.); m.napolitano@istitutotumori.na.it (M.N.); g.rea@istitutotumori.na.it (G.R.); l.portella@istitutotumori.na.it (L.P.); s.santagata@istitutotumori.na.it (S.S.); 2 Institute of Biostructures and Bioimaging of the National Council of Research, 80145 Napoli, Italy; antonella.zannetti@cnr.it (A.Z.); adegreco@unina.it (A.G.); sandralbanese@gmail.com (S.A.); 3 Interdepartmental Center of Veterinary Radiology, University of Naples Federico II, 80131 Naples, Italy; 4 Pharmacy Unit, Istituto Nazionale Tumori IRCCS “Fondazione G. Pascale”, 80131 Napoli, Italy; p.maiolino@istitutotumori.na.it; 5 Department of Pathology, Istituto Nazionale Tumori IRCCS “Fondazione G. Pascale”, 80131 Napoli, Italy; giosue.scognamiglio@istitutotumori.na.it (G.S.); f.tatangelo@istitutotumori.na.it (F.T.); g.botti@istitutotumori.na.it (G.B.); 6 Department of Oncology, Istituto Nazionale Tumori IRCCS “Fondazione G. Pascale”, 80131 Napoli, Italy; s.tafuto@istitutotumori.na.it (S.T.); g.nasti@istitutotumori.na.it (G.N.); a.ottaiano@istitutotumori.na.it (A.O.); 7 Department of Advanced Biomedical Sciences, School of Medicine, University of Naples Federico II, 80131 Naples, Italy; pacerto@yahoo.com; 8 Department of Colorectal Surgery, Istituto Nazionale Tumori IRCCS “Fondazione G. Pascale”, 80131 Napoli, Italy; p.delrio@istitutotumori.na.it

**Keywords:** colorectal cancer, CXCR4/CXCL12 axis, EMT epithelial-to-mesenchymal transition, radiotherapy, chemotherapy, chemoresistance

## Abstract

The chemokine receptor CXCR4 is overexpressed and functional in colorectal cancer. To investigate the role of CXCR4 antagonism in potentiating colon cancer standard therapy, the new peptide CXCR4 antagonist Peptide R (Pep R) was employed. Human colon cancer HCT116 xenograft-bearing mice were treated with chemotherapeutic agents (CT) 5-Fluorouracil (5FU) and oxaliplatin (OX) or 5FU and radio chemotherapy (RT-CT) in the presence of Pep R. After two weeks, CT plus Pep R reduced by 4-fold the relative tumor volume (RTV) as compared to 2- and 1.6-fold reductions induced, respectively, by CT and Pep R. In vitro Pep R addition to CT/RT-CT impaired HCT116 cell growth and further reduced HCT116 and HT29 clonal capability. Thus, the hypothesis that Pep R could target the epithelial mesenchyme transition (EMT) process was evaluated. While CT decreased ECAD and increased ZEB-1 and CD90 expression, the addition of Pep R restored the pretreatment expression. In HCT116 and HT29 cells, CT/RT-CT induced a population of CD133+CXCR4+ cells, supposedly a stem-resistant cancer cell population, while Pep R reduced it. Taken together, the results showed that targeting CXCR4 ameliorates the effect of treatment in colon cancer through inhibition of cell growth and reversal of EMT treatment-induced markers, supporting further clinical studies.

## 1. Introduction

Colorectal cancer (CRC) is among the most frequently diagnosed malignancies in western countries [1]. Despite improved knowledge, diagnostics and screening, up to 30% of patients present with synchronous metastases [2], and 40–50% will eventually develop metastases after primary therapies for localized disease within 3 years from diagnosis [3]. Colonization of distant organs depends on acute changes in cellular attributes such as adhesion and migratory potential [4]. These features characterize the epithelial-to-mesenchyme transition (EMT) program in which epithelial cells gradually lose their morphological features (polarity, membrane adhesion, cell-to-cell contacts) to acquire spindle morphology [5]. Recent evidence identified some detrimental aspects of chemotherapy that could induce the EMT program, featured by apoptotic tolerance, resistance-genes expression, increased dissemination and stemness phenotype [5]. In colon cancer, low E-Cadherin (CDH1/ECAD) and high N-Cadherin significantly correlated with local infiltration, tumor stage and vascular invasion [6]. miR-125b regulates Wnt/β-catenin signaling and triggers EMT, conferring 5FU (5-fluorouracil) resistance [7], while chronic oxaliplatin (OX) induces chemoresistance through EMT [8,9]. The chemokine receptor CXCR4 is overexpressed in colon cancer [10], where it represents a poor prognostic factor [11,12,13]. The CXCL12/CXCR4 axis also activates the EMT program in colorectal cancer, targeting the Wnt/β-catenin pathway [7,14,15,16], and in multiple carcinomas such as pancreatic cancer [17], ovarian cancer [18] and non-small cell lung cancer (NSCLC) [19]. The aim of the study was to improve the efficacy of colon cancer standard therapy targeting CXCR4 with a new CXCR4 antagonistic peptide by reversing the EMT program [20].

## 2. Results

### 2.1. Peptide R Reduces HCT116 Tumor Growth and Improves Standard Chemotherapy Efficacy in HCT116 Xenograft

**Figure 1:**
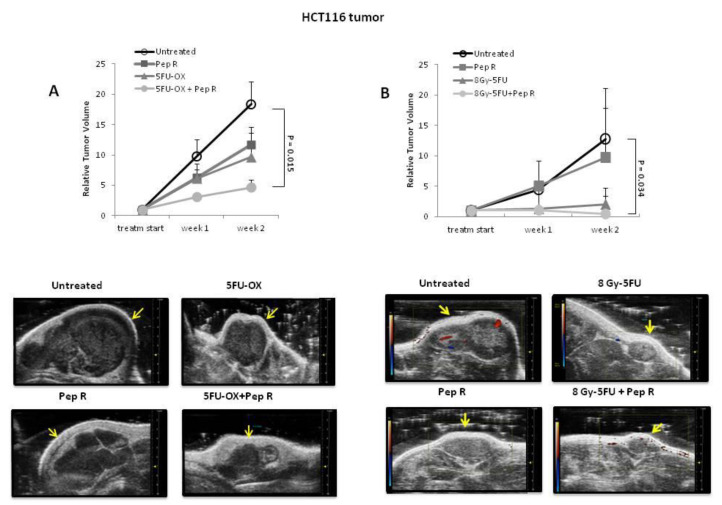
Peptide R reduces HCT116 tumor growth and improves the efficacy of radiotherapy chemotherapeutic agents (RT-CT) in colon cancer HCT116-xenograft. (**A Upper**): Relative tumor volumes ± SEM (6 animals /group). Significance *p* < 0.05. Repeated measures (RM) ANOVA with Bonferroni post hoc test and correction for multiple testing by Bonferroni’s adjustment. (**A Lower**): tumors high frequency ultrasound (HFUS) conducted at 14 days of treatment (scale bar on the right). (**B Upper**): relative tumor volumes ± SEM (6 animals Control and 4 animals for each treatment group). Significance *p* < 0.05. RM ANOVA with Bonferroni post hoc test and correction for multiple testing by Bonferroni’s adjustment. (**B Lower**): color doppler high frequency ultrasound (HFUS) representative images of HCT116 tumors (arrow) at 2 weeks of treatment; red spots identify blood flow entering the tumor while blue spots identify blood flow leaving the tumor. Three mice per group; one mouse per group represented.

The new peptide CXCR4 antagonist, Pep R, was associated with standard chemotherapy in colorectal cancer. Nude mice were subcutaneously (SC) injected with HCT116 cells and, when the tumor was well-established, the animals were treated with either 5-Fluorouracil (5FU) + Oxaliplatin (OX) (CT), Pep R, or both (5FU-OX + Pep R) (Figure S1A). After two weeks of treatment the mean relative tumor volumes (RTV) were 18.41 ± 6.04 for tumors in untreated mice, 9.67 ± 4.00 in CT-treated and 11.70 ± 2.89 in Pep R-treated mice, 2- and 1.6 fold less than untreated mice, respectively. The addition of Pep R to CT significantly improved the efficacy of chemotherapy, reducing RTV 4-fold (4.67 ± 1.17) (Figure 1A). To characterize the effect on tumor growth, mice were subjected to high frequency ultrasound (HFUS) after two weeks of treatment. In Figure 1A, lower panel, representative HFSU images of HCT116 tumors are reported, showing that Pep R also reduced tumor size at day 14 post treatment. Pep R activity on RT-CT was blunted by RT efficacy (Figure 1B). In Figure 1B lower panel, representative ultrasound color doppler HFUS images display different tumor sizes and pattern of vascularization at day 14 post treatment, with reduction in incoming flux in the treated tumors. Furthermore, a positron emission tomography (PET)/CT scan using ^18^F-FDG was conducted on three mice showing reduction in tracer uptake in RT-CT, and in Pep R treated tumors, consistent with reduced tumor metabolism as previously reported for CXCR4 antagonists [21] (Figure S2). These results suggest a direct effect of CXCR4 inhibition on colon cancer cell growth in accordance with previous evidence [22].

### 2.2. Peptide R Targeting CXCR4 Impairs Cell Growth and Potentiates In Vitro Chemoradiotherapy Sensitivity

**Figure 2:**
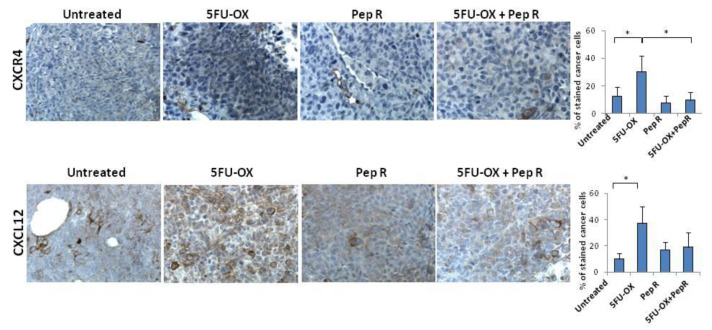
Peptide R modulates CXCR4 in CT treated HCT116 human colon cancer xenograft. Immunohistochemistry staining (200× magnification) for CXCR4 and CXCL12. Bar graph illustrating quantifications of IHC staining from collected tumors (means ± SD). A *p*-value < 0.05 (*) was considered statistically significant (Kruskal-Wallis test followed by Dunn’s multiple comparison). Percentage of stained cancer cells y axis (y-axis); treatments (x-axis).

To analyze the effect of Pep R on colon cancer cell growth, CXCR4 and CXCL12 expression were evaluated in HCT116 tumors. CXCR4 was localized at the cell membrane and/or cytoplasm of cancer cells and its expression was heterogeneous within the tumors, probably in relation to hypoxic and necrotic areas [23] (Figure S3). As shown in Figure 2, CT increased CXCR4 expression in HCT116 tumors partially reversed by the addition of Pep R. Pep R-dependent CXCR4 reduction was also confirmed at the RNA level (Figure S4).

**Figure 3:**
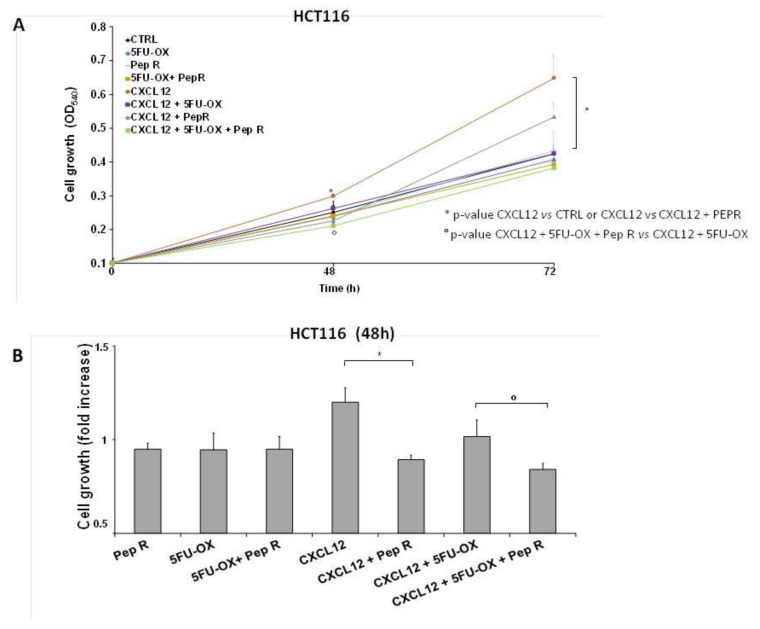
Peptide R impairs cell growth and potentiates in vitro CT/RT-CT efficacy. HCT116 were grown in the presence of CXCL12 (100 ng/mL), 5FU-OX (3.5–4.5µM. respectively), Pep R (10 µM). (**A**) Graph showed showing Pep R (10 µM) enhanced significantly 5-FU-OX effect at 48 h (**B**). Bars depict mean ± SD of three independent experiments. *p*-value < 0.05 (*) was considered statistically significant (Kruskal-Wallis test followed by Dunn’s multiple comparison).

The ligand CXCL12 significantly increased in the CT-treated tumor and decreased with the combined treatment with Pep R. This could reflect an autocrine loop previously demonstrated in colon cancer cells in which the CXCR4-CXCL12 axis controls cell growth [22]. In vitro HCT116 cell growth was evaluated in the presence of CT and Pep R. CXCL12 significantly induced cell growth while Pep R decreased it (Figure 3). As shown, in Figure 3B the addition of Pep R at 48 h reduced the CXCL12-dependent HTC116 cell growth as well as improved the efficacy of CT. 

**Figure 4:**
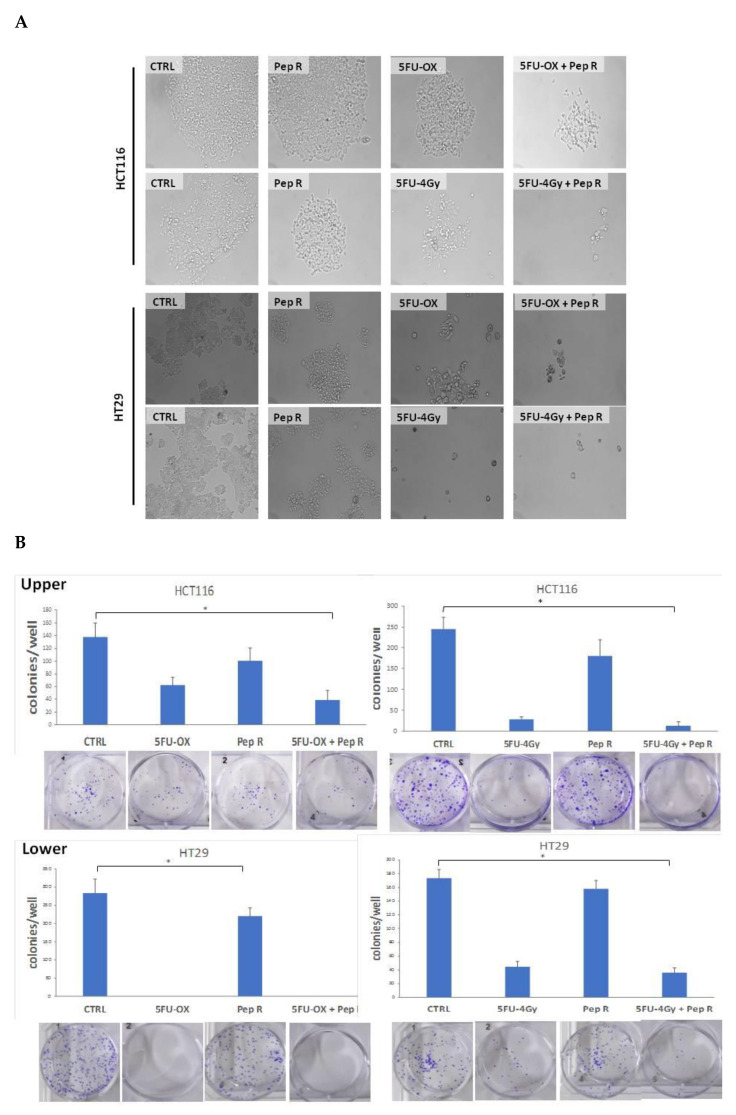
Peptide R rescued CT/ RT-CT sensitivity. (**A**) Microphotograph of HCT116 (**A upper panel**) and HT29 (**A lower panel**), untreated cells and after treatment with CT/RT-CT plus Pep R. (10× magnification); (**B**) HCT116 (**B Upper**) and HT29 (**B Lower**) cell line were evaluated for clonal capability. Representative well plate images for each treatment are shown below the bar graph. Bar graphs show clone number (mean ± SD) for each treatment. A *p*-value < 0.05 (*) was considered statistically significant (Kruskal-Wallis test followed by Dunn’s multiple comparison).

In Figure 4A, a representative micrograph of cultured cells showed HCT116 and HT29 cell growth reduction in the presence of CT and RT-CT, improved by the addition of Pep R. In Figure 4B, the HCT116 and HT29 clonal efficiency impairment due to CT/RT-CT was further inhibited by the addition of Pep R.

### 2.3. Peptide R modifies CT/RT-CT-Induced EMT markers in HCT116 Xenograft Tumors

**Figure 5:**
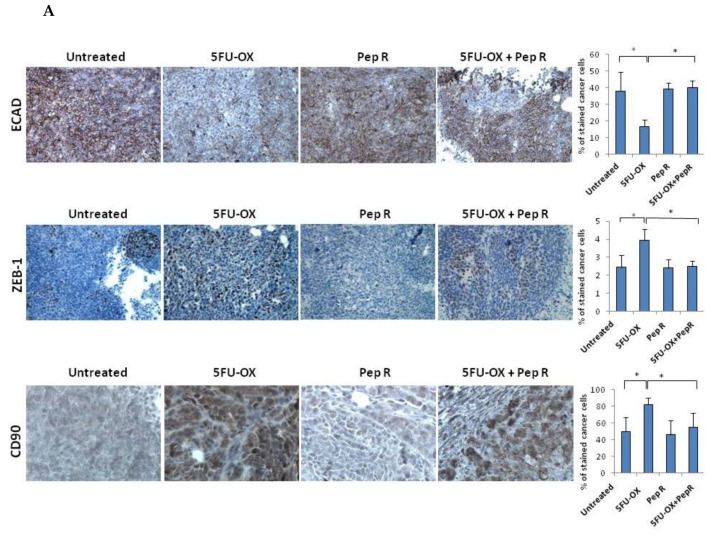

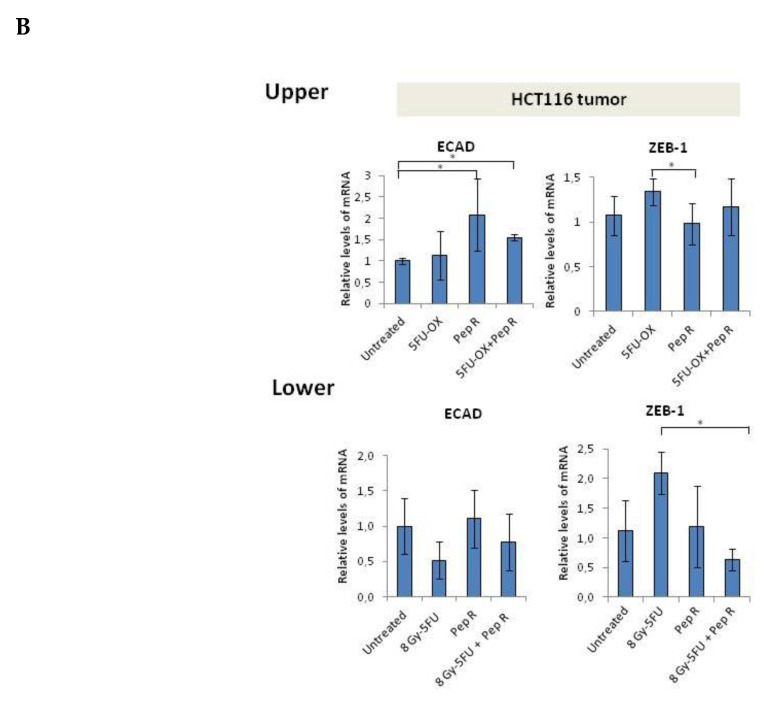
In vivo Peptide R modulates EMT-inducing genes in HCT116 human colon cancer xenograft. (**A**) Representative immunohistochemistry staining (200× magnification) of membranous E-cadherin, nuclear ZEB1 in tumor cells of invasive tumor regions; cytoplasmic CD90 (brown staining). Bar graph illustrating quantification of IHC staining from collected tumors (means ± SD)**.** A *p*-value < 0.05 (*) was considered statistically significant (Kruskal-Wallis test followed by Dunn’s multiple comparison). Percentage of stained cancer cells y axis (y-axis); treatments (y-axis). (**B**) RNA expression for ECAD and ZEB-1 expression in HCT116 tumors. Bar graph illustrating relative mRNA expression levels (qRT-PCR) of indicated genes (means ± SD). The 2^-ΔΔCT^ method was used as a relative quantification. Triple determination each point/gene were performed. A *p*-value < 0.05 (*) was considered statistically significant.

Since CXCR4 was reported to activate the EMT program in colorectal cancer [7,14,15,16], and chemotherapy to induce chemoresistance through the EMT program [5], EMT transition was evaluated as a possible Pep R mechanism of action. In Figure S5 in silico correlation from 275 colon adenocarcinoma (COAD) plus 91 rectum adenocarcinoma (READ) TCGA dataset RNA-Seq displayed the association between the CXCR4/CXCL12 axis and EMT master regulators in colorectal cancer. As shown in Figure 5A, in HCT116 xenografts CT reduced ECAD expression whereas the addition of Pep R to CT recovered it. Conversely, in the same tumor, CT increased ZEB-1 and CD90 levels while the combined treatment with Pep R reverted the expression of the mesenchymal markers. Interestingly, Pep R also reduced PD-L1 expression -CT-induced, as shown in Figure S6. Furthermore, as shown in Figure 5B, ECAD mRNA levels were reduced in CT/RT-CT- treated tumors while ZEB-1 expression was increased after treatments. Although to a different extent, Pep R addition reverted the effect of conventional therapies on mesenchymal markers, suggesting a role for CXCR4 in controlling EMT marker expression. Pep R increased ECAD expression by 2.08-fold (*p* = 0.04) and 1.53 fold in combination with 5FU-OX (*p* = 0.007), while it reduced ZEB-1 mRNA levels by 1.36 fold (*p* = 0.006) (Figure 5B Upper). Similar results were reported in the RT-CT treated mice (Figure 5B Lower). Altogether, the data indicate that ECAD and ZEB-1 are important for HCT116 chemotherapy-induced cellular plasticity, and that these mesenchymal features are modulated by CXCR4 inhibition [19].

### 2.4. Peptide R modulates CT/RT-CT-Induced EMT markers in Human Colon Cancer Cells

**Figure 6:**
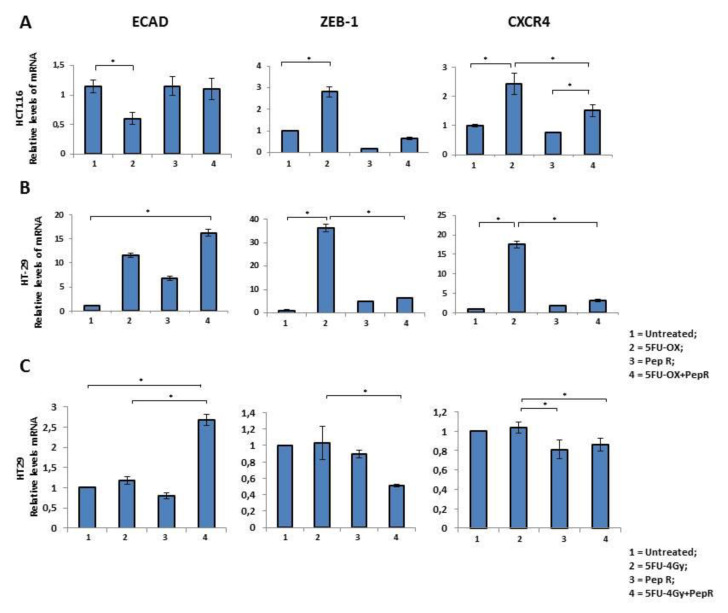
In vitro Peptide R modulates CT-inducing EMT markers. HCT116 (**A**) -2HT9 (**B**). In vitro Peptide R modulated CT/RT-CT EMT-inducing genes in HT29 (**C**). Bar graph illustrating relative mRNA expression levels (qRT-PCR) of indicated genes (means ± SD). The 2^-ΔΔCT^ method was used as a relative quantification strategy data analysis. Triple determination for each point/gene were performed. *p*-value < 0.05 (*) (Kruskal-Wallis test followed by Dunn’s multiple comparison).

EMT markers were evaluated in HCT116 and HT29 cells. As shown in Figure 6 the effect of CT/RT-CT +/- Peptide R was analyzed on the expression of ECAD, ZEB-1 and CXCR4. In HCT116 cells, CT reduced ECAD and significantly induced ZEB-1 and CXCR4 (Figure 6A). Interestingly, the effect of CT on ZEB-1 and CXCR4 expression was reversed by the addition of Pep R. In HT29 cells, CT induced ECAD according to a previous report [24], and the effect was further improved by Pep R. In the same cell line, Pep R reduced ZEB-1, and CXCR4 expression was significantly induced by CT treatment (Figure 6B)**.** Similarly, RT-CT+ Pep R treatment of HT29 cells significantly increased ECAD expression while decreasing ZEB-1 and CXCR4 mRNA levels (Figure 6C). 

**Figure 7:**
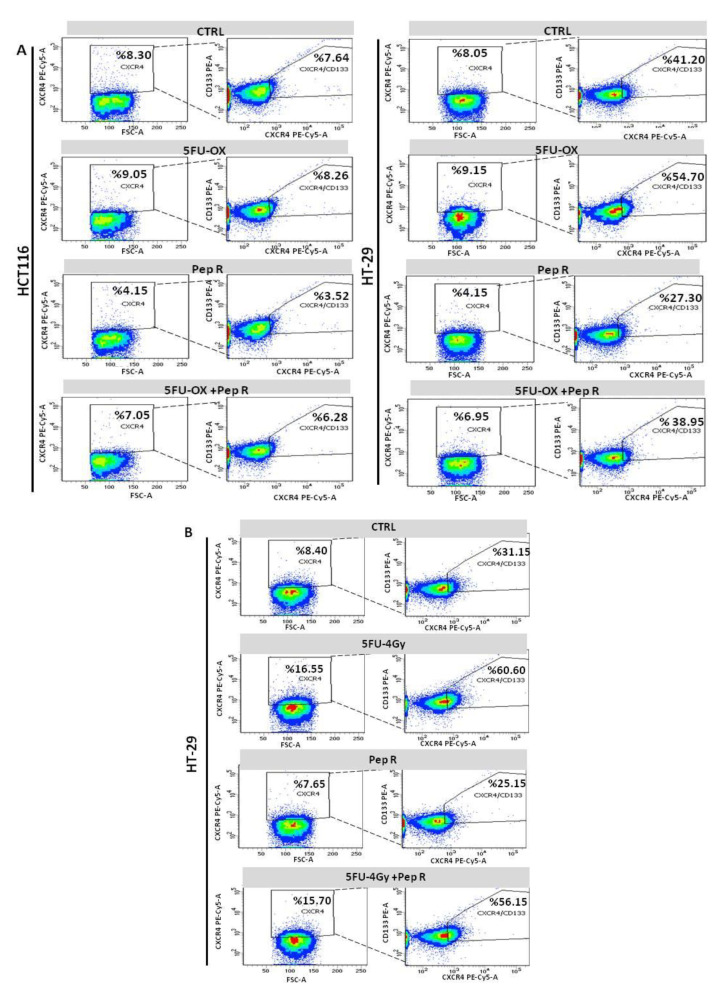
Pep R reduces CXCR4+CD133+ cancer-stem-cell component CT/RT-CT-induced. Flow cytometry analysis for CXCR4 and CD133 expressing cells. HCT116 (left panel) and HT29 (right panel) cells CT treated plus Pep R (**A**)**.** HT29 RT-CT treated plus CXCR4 plus Pep R (**B**)**.**

Noteworthy, both CT (Figure 7A) and RT-CT (Figure 7B) selected a stem cell population (CXCR4+CD133+) reduced by Pep R addition.

## 3. Discussion

The newly developed CXCR4 antagonist, Peptide R (Pep R), reduced the growth and improved the efficacy of conventional chemo (CT) or chemo-radiotherapy (RT-CT) in a HCT116 human colon cancer model. This effect relied on reduction of cell growth and modulation of the mesenchymal-stem cell transition, CT/RT-CT induced [25,26]. The CXCR4 receptor and relative transcripts decreased with Pep R treatment according to a previous report [27]. Previous evidence demonstrated that Pep R reduced the CXCL12 mediated internalization in HCT116 human colon cancer cells [28], possibly adding this effect to transcriptional downregulation. Motixafortide/BL-8040, a short synthetic peptide CXCR4 antagonist, also inhibited CXCR4 internalization [29]. Cancer cells with mesenchymal features shape the phenotype and the activity of tumor-associated immune cells, which in turn can regulate cancer cells’ EMT, releasing soluble mediators, regulating chemoresistance [30] and tumor immune escape [31]. Herein ECAD and ZEB-1 were modulated by CT/RT-CT, resulting in ECAD reduction and promoting ZEB-1 and CD90/THY-1 expression. Among proteins featuring EMT, CD90 was proposed as a marker of circulating tumor cells [32]. CD90 is a glycophosphatidylinositol-anchored cell surface protein identified as a cancer stem cell marker in glioma, liver, gastric cancer and basal-like triple negative breast cancer patients [33]. Herein, CD90 was induced by CT in HCT116, while Pep R reduced it concomitantly to ZEB-1 and to the normalization of ECAD. CXCR4 targeting reduces CD90^+^ fractions, increasing the transition toward the epithelial state as previously reported in human spermatogonia stem cells [34]. Recently a pan-cancer EMT signature was derived from 11 cancer types including breast, lung, colon, ovarian and bladder cancers [35]. It comprised immune checkpoints PD1, PD-L1, CTLA4, OX40L and PD-L2, and the most mesenchymal EMT scores, highlighting the correlation between EMT and immune resistance [36]. Moreover, the immune checkpoints B7-H3/CD276 and OX40 were found to be significantly co-expressed with core EMT genes, TGFB1, CXCR4, IL10, and IL6 [36] on tumor microenvironment as vascular endothelial and myeloid cells [37].

Several reports connected CXCR4 expression, EMT and metastasis in colon cancer [7,16]. CXCR4 promoted EMT and infiltration of myeloid-derived suppressor cells and macrophages in colitis-associated cancer [16]. Tumor-associated macrophages CD206^+^ TAMs, which infiltrated at the invasive front, were correlated with CXCR4 expression and liver metastasis. Several miRNAs (miR-25-3p, miR-130b-3p, miR-425-5p) upregulated in CRC cells by the CXCL12/CXCR4 axis could be transferred to macrophages via exosomes inducing M2 polarization of macrophages that promoted cancer metastasis by enhancing EMT [38,39]. Moreover, the CXCR4 antagonist Nef-M1, inducing E-CAD and decreasing the mesenchymal signature markers vimentin, fibronectin, and p-GSK-3β, reverts EMT in colon and breast xenograft models [15].

Standard therapy may present some detrimental aspects such as tissue damage, hypoxia and cancer cell apoptosis through the release of proinflammatory cytokines such as TNF-alpha, granulocyte-colony stimulating factor (G-CSF), CXCL12, CCL2-4 and ICAM1 [40]. At the primary tumor site, chemotherapy induced selection of specific clones possessing intrinsic resistance properties, stemness features, mesenchymal phenotype and biological aggressiveness [5]. Herein, the CXCR4 antagonist sensitized cells to therapy effect, reducing the development of mesenchymal, stem cells, CXCR4 expressing [18,41]. Pep R could also target CXCR4+ expressing stromal cells, further impairing mechanisms of EMT and chemoresistance. Targeting the CXCR4–CXCL12 axis exerts activity on TME, also reverting the tolerogenic polarization of immunosuppressive cells such as regulatory T cells (Treg) [42]. Improvement in antiPD1 function was revealed in murine colon cancer models by combined treatment with Pep R [43] and NOXA-012, CXCL12-targeting antagonists [44].

## 4. Materials and Methods

### 4.1. Ethics statement

The study was approved by Istituto Nazionale per lo Studio e la Cura dei Tumori, “Fondazione G. Pascale” IRCCS-Italy Ethics committee (CE 689-24/10/2007).

### 4.2. Peptide R Modulates Cell lines

Human colon cancer cells, HCT116, HT-29, obtained from the National Cancer Institute’s Developmental Therapeutics program (NCI DTP) were maintained in RPMI 1640 media (Invitrogen, San Diego, CA, USA) containing 10% fetal bovine serum.

### 4.3. Clonogenic Assay

HCT116 and HT29 cell lines were treated with fluoruracil and oxaliplatin chemotherapy (5FU-OX) (1.75 μM 5-Fluoruracil and 5 μM Oxaliplatin) alone and in combination with Pep R (10 μM). Further radiochemotherapy was tested: 5-Fluoruracil (5FU) (1.75 μM) and Pep R for three days. Then, cells subjected to 5FU and Pep R combination were irradiated at a dose of 4 Gy. After ~6 h, HT29 and HCT116 cells were plated (700 cells/well) and incubated for 7 days. The images were assessed by live imaging using a Zeiss AxioScope light microscope (City, state abbreviation if USA/Canada, Country). Plates were then stained with crystal violet and colonies consisting of 50 or more cells were manually counted.

### 4.4. Flow Cytometer

FACS analysis was conducted on a FACS Canto II 6-colour flow cytometer with FACs Diva software (BD Biosciences, San Jose, CA USA). Surface staining was performed in the dark for 30 min at 4 °C in staining buffer using antihuman CD274/PD-L1 (B7-H1)-PE-Vio615, antihuman CD90 FITC Antibody (Miltenyi Biotech, Bergisch Gladbach, Germany), CD133 antibody PE conjugated antihuman CD133/2 (AC133) (Miltenyi Biotech), CXCR4 PE-Cy5 conjugated antihuman CD184 (BD Biosciences, San Jose, CA USA), Isotype control antibody PE Mouse IgG2B (R&D systems, Minneapolis, MN, USA or Miltenyi Biotech). Cells were washed and stained with a viability dye (eFluorTM780, eBioscience, Thermo Fisher Scientific, Waltham, MA USA) prior to fixation procedures with 2% paraformaldehyde.

### 4.5. Animal Tumor Models and Treatments

All experimental procedures complied with the European Communities Council directives (2010/63/EU) in accordance with National Institutes of Health (NIH) recommendations. The present study was approved by the Italian Ministry of Health (2013/0100808). All efforts were made to minimize animal suffering and the number of animals necessary to produce reliable results. Six-weeks-old female Hsd: Athymic Nude-Foxn1nu mice (*n* = 42) (ENVIGO RMS S.R.L., Udine, Italy) were housed in a specific pathogen-free facility for one week before the beginning of the experiments. A priori power analysis was conducted using the Gpower program (G*Power software package, version 3.1.4, Heinrich-Heine-Universität Düsseldorf, Düsseldorf, Germany). Mice were injected subcutaneously into the left flank with 2.5 × 10^6^ HCT116 cells suspended in PBS in a final volume of 200 µL. When the tumor was measurable, intraperitoneal (ip) treatment started with 5-Fluorouracil (5FU) (30 mg/Kg); oxaliplatin (OX) (4.2 mg/kg); Peptide R (Pep R) (5 mg/Kg) [20,45]; Radiotherapy (a single fraction of 8 Gy) (scheme of treatments is shown in Figure S1). The tumor volume was measured with a caliper and derived by the formula: Tumor Volume (TV) (mm^3^) = L × W^2^/2; where L is the length and W is the width. The individual relative tumor volume (RTV) was calculated using the following formula: RTV = Vx/V1 where Vx is the caliper-derived volume in mm^3^ at a given time and V1 at the start of treatment. The mean ± SEM were calculated for all the groups. We analyzed response time trends recorded for different treatments by comparing means at each time point.

### 4.6. Non-Invasive High-Frequency Ultrasound (HFUS) Imaging of Xenografts

A high-frequency ultrasound (HFUS) system (VEVO 2100, FUJIFILM VisualSonics, Inc., Toronto, ON, Canada), mounting a 40 MHz transducer (MS 550 D, FUJIFILM VisualSonics, Inc.) was used to evaluate tumor growth. The HFUS evaluations were conducted in anesthetized mice (2% isoflurane in 100% oxygen at 0.8 L/min). Each mouse was placed in the right lateral recumbence on a dedicated small animal table (VEVO Imaging Station 2, FUJIFILM VisualSonics, Inc.). Brightness (B-) mode and color-doppler mode images were obtained for each tumor in two orthogonal planes, i.e., the trans-axial and the sagittal planes.

### 4.7. Imaging Studies with ^18^F-FDG Small-Animal PET/CT

^18^F-FDG and a small-animal Positron Emission Tomography/Computerized Tomography scanner (eXplore Vista Pre-Clinical PET Scanner GE Healthcare, Little Chalfont, Buckinghamshire, UK) were used. After fasting 8 h, animals received 200 microCi (7.4 MBq) of ^18^F-FDG by injection into the tail vein. Animals were anesthetized using 2% isoflurane in 100% oxygen at 0.8 L/min and subjected to a PET/CT scan 60 min after injection. Body temperature of the animals was held constant during tracer biodistribution, positioning mice on a heating pad. Briefly, one bed position, including the tumor, was scanned (axial field of view, 68 mm) and CT images were acquired with the X-ray source set at 35 kVp and 200 mA for 10 min followed by 20 min PET acquisition. After acquisition, the images were reconstructed through a combination algorithm based on Fourier rebinning followed by 2-dimensional iterative image reconstruction with ordered-subsets expectation maximization. PET and CT images were automatically co-registered, and fusion images were obtained [46,47].

### 4.8. Real-Time Polymerase Chain reaction (PCR).

RNA was extracted from fresh tumor tissues and cell lines with TRIzol Reagent (Invitrogen, Carsbald, CA, USA) following the manufacturer’s instructions. For extraction of RNA from FFPE tumor tissue, *n* = 10 sections of 5-µm were cut from each archival block. Paraffin was removed by xylene extraction followed by ethanol washes. RNA was isolated from tissue slices using the FFPE RNA Purification Kit (Norgen Biotek Corp., Thorold, ON, Canada), Quantitative real-time PCR was performed using SYBR Green PCR Master Mix (Applied Biosystems, Foster CA, USA) and data were collected and quantitatively analyzed on an QuantStudio™ 5 Real-Time PCR System with 2^−ΔΔCt^ method. Primers sequences for ECAD, ZEB-1, and CXCR4 are detailed in Table S1. Relative mRNA expression was normalized with β-actin (ACTB) gene expression. The primer pairs were subjected to a specificity checking process through the Primer-BLAST publicly available tool . 

### 4.9. Immunohistochemistry

FFPE tissue blocks derived from surgically-collected tumor samples were obtained. After heat-induced epitope retrieval (HIER) the sections were incubated with following antibodies: E (epithelial)-cadherin Antibody (M3612 DAKO (NCH-38), 1:75 dilution, CXCR4 Antibody (Antihu-CXCR4 antibody; UMB2 ABCAM; 1:100 dilution, pH6), and ZEB1 Antibody (HPA027524, Atlas Antibody, 1:350 dilution, pH 6) CXCL12 (1:50 dilution clone MAB350 pH 6, R&D Systems). Rabbit antihuman mAb PD-L1 (1/300 dilution, pH 8 (E1L3N^®^) XP^®^), CD90/Thy1 Antibody (1/100 dilution, pH 6, EPR3133, abcam) and with appropriate secondary antibody HRP linked (DAKO, Cambridgeshire, UK) for 30 min. The staining was based on the rate of stained cancer cells for HPF field (400× magnification), at least 10 HPF/slide in at least 5 in areas; stained sections were independently evaluated by three expert pathologists/researchers (FT/GS and CD) blind to initial assessments.

### 4.10. In Silico Studies

Correlation between CXCR4-CXCL12 axis and EMT regulator genes in CRC was performed by the online tools GEPIA. GEPIA is a web server for analyzing the RNA sequencing expression data of 9736 tumors and 8587 normal samples from the TCGA and the GTEx projects using a standard processing pipeline.

### 4.11. Statistical Analysis

SPSS software (version 20.0, SPSS, Inc., Chicago, IL, USA) was used for statistical analysis. The continuous variables were compared using an unpaired Student’s *t-*test or a Mann-Whitney U test if the variables were not normally distributed. A Kruskal–Wallis test followed by Dunn’s multiple test comparison was used to determine significantly different groups. A repeated measures ANOVA with Bonferroni post hoc test and correction for multiple testing by Bonferroni’s adjustment was used to determine treatment effect over time. *p* < 0.05 was considered statistically significant.

## 5. Conclusions

Taken together our preclinical results show that a newly developed CXCR4 antagonist Pep R is able to improve standard therapy efficacy targeting cell growth and mesenchymal transition, endorsing further clinical studies for association of CXCR4 antagonists plus standard therapy in colorectal cancer.

## Supplementary Materials

The following are available online at https://www.mdpi.com/2072-6694/12/7/1952/s1; Figure S1: Treatment schedule of standard radiochemotherapy in HCT116 human colon xenograft, Figure S2: ^18^F-FDG uptake decreased in 5-FU-RT treated tumors and in Pep R treated tumors, Figure S3: CXCR4 expression increased in tumor xenografts compared to cells growing in culture, Figure S4: Pep R significantly reduced CXCR4 gene expression, Figure S5: CXCR4 and CXCL12 expression significantly correlates with mesenchymal markers in colorectal cancer, Figure S6: In vivo and in vitro Peptide R modulated 5FU-OX induced PD-L1 in HCT116 human colon cancer, Table S1: Primer sequences for SYBR Green RT-qPCR.

## Abbreviations

CXCR4	C-X-C chemokine receptor type 4
SDF1/CXCL12	stromal cell-derived factor 1/ C-X-C motif chemokine 12
CRC	Colorectal cancer
OX	oxaliplatin
5FU	Fluorouracil
CT	5-Fluorouracil and Oxaliplatin chemotherapy
RT-CT	Radiotherapy plus–5FU
EMT	epithelial-to-mesenchymal transition
CDH1/E-CAD	E-Cadherin
RTV	relative tumor volume
TV	tumor volume
Pep R	peptide R
CSC	cancer stem cell
CTC	circulating tumor cell
