# Supplementary material for: New CXCR4 Antagonist Peptide R (Pep R) Improves Standard Therapy in Colorectal Cancer
Source: Cancers (Basel). 2020 Jul 18;12(7):1952. doi: 10.3390/cancers12071952 (PMC7409147; doi:10.3390/cancers12071952)
Supplement: Supplementary file 1 [file cancers-12-01952-s001.pdf]

Supplementary Material: New CXCR4 Antagonist Peptide R (Pep R) Improves Standard Therapy in Colorectal Cancer

Crescenzo D'Alterio, Antonella Zannetti, Anna Maria Trotta, Caterina Ieranò, Maria Napolitano, Giuseppina Rea, Adelaide Greco, Piera Maiolino, Sandra Albanese, Giosuè Scognamiglio, Fabiana Tatangelo, Salvatore Tafuto, Luigi Portella, Sara Santagata, Guglielmo Nasti, Alessandro Ottaiano, Roberto Pacelli, Paolo Delrio, Gerardo Botti and Stefania Scala

Table S1. Primer Sequences for SYBR Green RT-qPCR.

Primer	Sequence (5′ → 3′)
ECAD-FWD	GTCAGTTCAGACTCCAGCCC
ECAD-REV	AAATTCACTCTGCCCAGGACG
ZEB-1-FWD	AAGAAAGTGTTACAGATGCAGCTG
ZEB-1-REV	CCCTGGTAACACTGTCTGGTC
CXCR4-FWD	TGAGAAGCATGACGG
CXCR4-REV	AGGGAAGCGTGATGA
ACTB-FWD	AAATCTGGCACCACACCTTC
ACTB-REV	GGGGTGTTGAAGGTCTCAAA

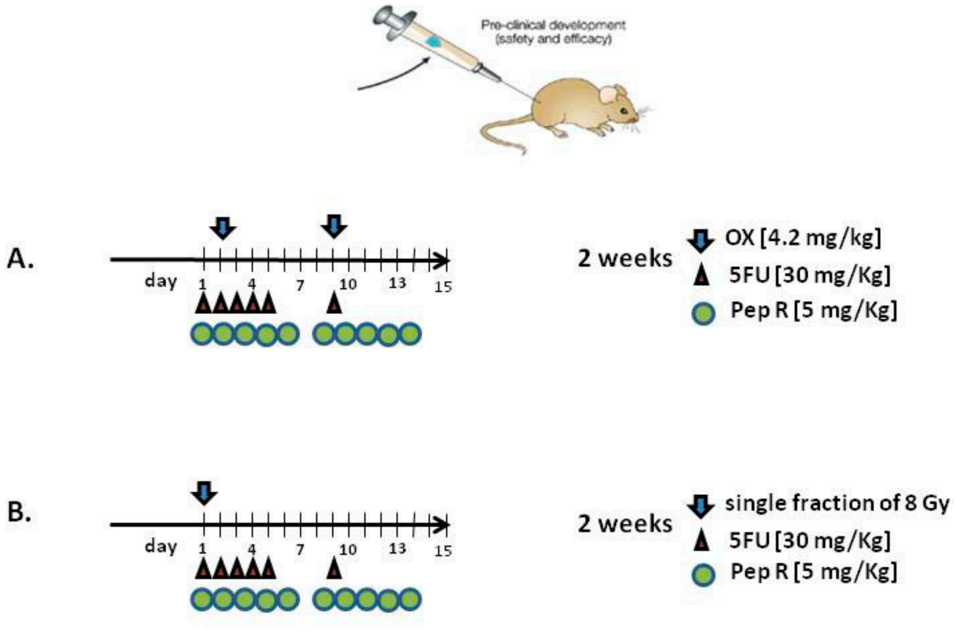

Figure S1. Treatment schedule of standard Radio-Chemotherapy in HCT116 human colon xenograft. Athymic mice were subcutaneously (s.c.) injected with HCT116 human colon cancer cells. Tumor bearing mice were treated as scheduled: 5-Fluorouracil (5FU) [30 mg/Kg daily 5 days for first week and once on day 9 for second week]; oxaliplatin (OX) [4.2 mg/kg once week on day 2 and day 9]; Peptide R (Pep R) 5 mg/Kg 10 days 5 days per week for two consecutive weeks (6 animals /group). Radio (once on day 1 a single dose of

6 MV X ray of a linear accelerator by a single fraction of 8 Gy) (6 animals Control and 4 animals for each treatment group).

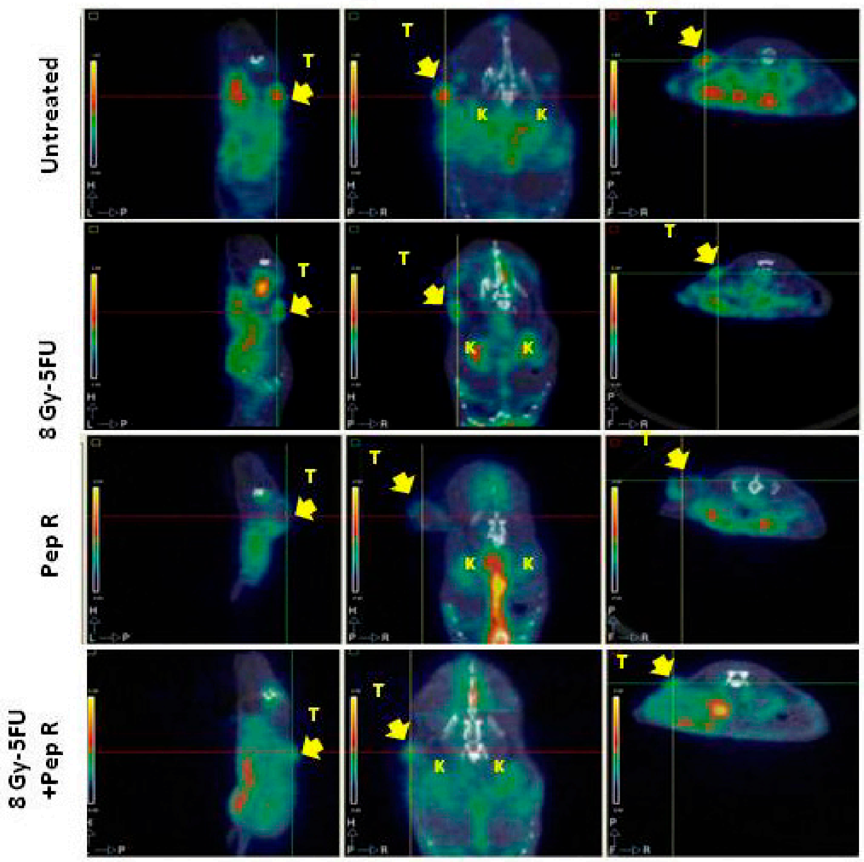

Figure S2. ^{18}F-FDG uptake decreased in 5-FU-RT treated tumors and in Pep R treated tumors. Representative sagittal. coronal and axial fusion images of small-animal PET/CT studies performed with ^{18}F-FDG. HCT116 tumors (yellow arrow and T) and kidneys (K) was annotated.

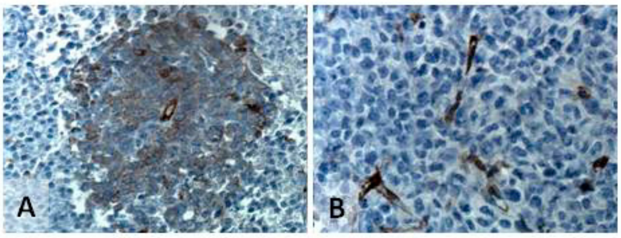

Figure S3. CXCR4 expression increased in tumor xenografts compared to cells growing in culture. CXCR4 expression increased in tumor xenografts for the presence of hypoxic and necrotic area within the tumor. (**A**) (400× magnification) CXCR4 expression negative in tumor cells but expressed by vascular endothelial and myeloid cell in tumor microenvironment (**B**) (400× magnification).

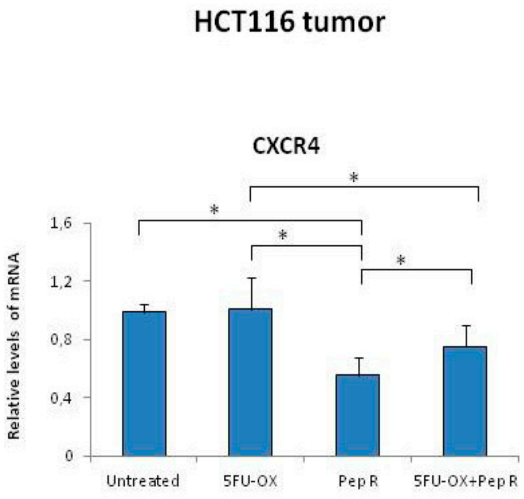

Figure S4. Pep R significantly reduced CXCR4 gene expression. Bar graph illustrating relative CXCR4 mRNA expression levels (qRT-PCR) (means ± SD) in HCT116 tumors. The $2^{-\Delta\Delta CT}$ method was used as a relative quantification strategy data analysis. RT-PCR for Fluorouracil (5FU) and oxaliplatin (OX) was performed on tumor sample from 6 animals /group. Triple determination each point /gene were performed. A *p*-value <0.05 (*) was considered statistically significant (Kruskal-Wallis test followed by Dunn's multiple comparison).

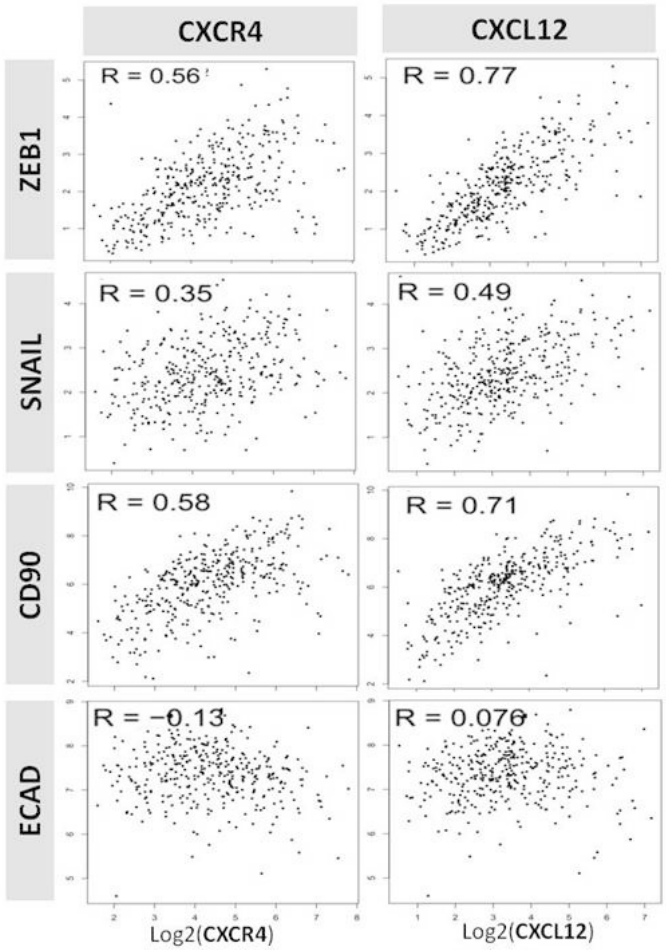

Figure S5. CXCR4 and CXCL12 expression significantly correlates with mesenchymal markers in colorectal cancer. In silico Spearman Correlation analysis from 275 colon adenocarcinoma (COAD) plus 91 rectum

adenocarcinoma (READ) TCGA dataset RNA-Seq. the association between the CXCR4/CXCL12 axis and EMT master regulators in colorectal cancer. GEPIA uses the non-log scale fold-change for calculation and use the log-scale, log2(gene fold-change), axis for visualization of gene expression.

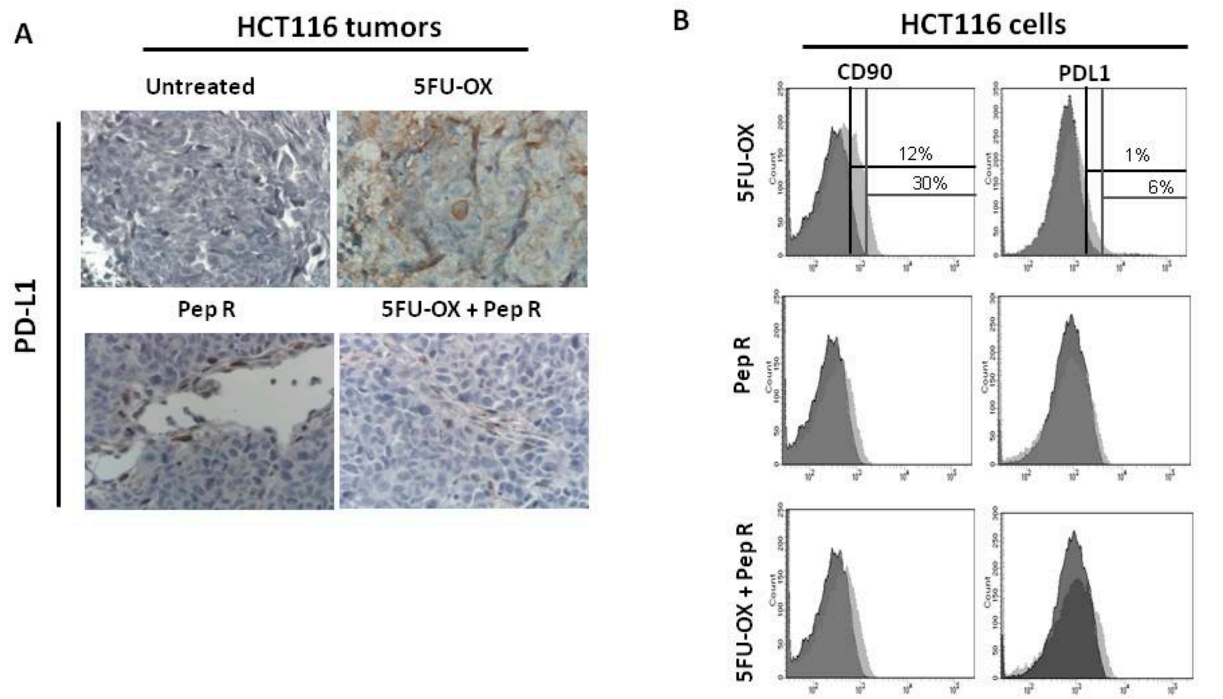

HCT116	% CD90	% PDL1
Untreated	12	1
5FU-OX	30	6
Pep R	7	3
5FU-OX + Pep R	15	4

Figure S6. In vivo and in vitro Peptide R modulated 5FU-OX induced PD-L1 in HCT116 human colon cancer. Representative immunohistochemical stainings (200x magnification) PD-L1 showing membranous immunoreactivity. PD-L1 positive cancer cells were reported on 3/6 collected tumor subjected to 5FU-OX treatment. none of the tumors belonging to other groups showed PD-L1 expression **(A).** Peptide R reverted in vitro Chemotherapy -induced CD90 and PD-L1 in HCT116 human colon cancer cells. Flow cytometry analysis for CD90 and PD-L1 in HCT116. Untreated control cells that express the antigen (CD90 or PD-L1) are plotted in dark grey. CD90 or PD-L1 expression in response to treatment are plotted in light grey **(B)**.
